# Construction and Validation of a Frailty Index in Primary Care in Italy: The Health-Search Frailty Index

**DOI:** 10.1093/geroni/igab046.2047

**Published:** 2021-12-17

**Authors:** Davide Vetrano, Alberto Zucchelli, Graziano Onder, Roberto Bernabei, Laura Fratiglioni, Alessandra Marengoni, Francesco Lapi

**Affiliations:** 1 Karolinska Institutet, Solna, Stockholms Lan, Sweden; 2 Università degli Studi di Brescia, Karolinska Institutet, Stockholms Lan, Sweden; 3 Italian National Institute of Health, Rome, Lazio, Italy; 4 Catholic University of Rome, Rome, Lazio, Italy; 5 Karolinska Institutet 17175, Stockholms Lan, Sweden; 6 University of Brescia, Brescia, Lombardia, Italy; 7 Health Search, Florence, Toscana, Italy

## Abstract

Recognizing frailty in primary care is important to implement personalized care pathways and for prognostication. The aim of this study was to build and validate a frailty index based on routinely collected primary care data in Italy. We used clinical data from 308,280 Italian primary care patients 60+ with at least 5 years of follow-up, part of the Health Search Database. A heuristic algorithm was used to select the deficits to be included in a highly performant frailty index. The fitness of the index was assessed through the c-statistics derived by survival models. Results were externally validated using the Swedish National Study on Aging and Care in Kungsholmen (SNAC-K). After testing 3.4 million of deficits combinations, 25 deficits were selected to be included in the Health Search Frailty Index (HS-FI). After adjusting by sex, age and geographical area, the HS-FI was associated with 5-year mortality (HR per 0.1 increase 1.99; 95%CI 1.95-2.02) and hospitalization rate (HR per 0.1 increase 1.25; 95%CI 1.23-1.27). In the external validation cohort, HS-FI independently predicted mortality, hospitalization, incident disability, incident dementia, and incident falls. This is the first frailty index built following a data-driven approach, using national representative primary care data. The implementation of such tool – derived by routinely collected data – in primary care software will ease the prompt, comparable and reliable recognition of frailty at the population level.

